# The Checking List Case Book

**Published:** 1896-05

**Authors:** 


THE CHECKING LIST CASE BOOK, by Maro F. Underwood, M. D., 924 Geary Street, San Francisco, California. Folio, 200 pages; price, $3.00. 400 pages, price, $5.00, postage prepaid.

This is a folio volume, with pages measuring sixteen inches by twelve. It is intended to assist in studying difficult cases, and is well adapted for the purpose. The editor of this journal has been making use of it for some months, with a view to testing its value before recommending it to the profession.

To enable the reader to understand its arrangement, we may thus explain it:

On opening the book we observe that the left-hand page is differently ruled from the right.

The left-hand page is divided into two columns, for writing down the symptoms of the patient. At the top of the page are headings with blank spaces, for inserting name, address, age, weight, and other necessary items of information.

The right-hand page is divided into four columns. On the left-hand borders of these columns are arranged the names of the remedies of the homœopathic materia medica in alphabetical order. The rest of the space in these columns is ruled off into very narrow *sub*-columns of about a quarter of an inch in width. In these sub-columns are placed letters of the alphabet from A to R, in a line running across from the name of the remedy.

In using the book, the main symptoms of the case are written down on the left-hand page, and, instead of being numbered, are marked each with a letter of the alphabet from A to R.

On studying the case when a symptom—say "D"—is found under any particular remedy, say Corallium-rumbrum, that remedy is hunted out in the right-hand page, the letter D is sought, and a dash marked under it. If any other remedies have this same symptom they too are marked. Proceeding in this way, we finally have all the symptoms accounted for under the different remedies. A glance of the eye serves to show which one of the remedies has the greatest number of these dashes opposite to it. This remedy, so indicated, is selected for administration to the patient. When the next report from the patient, after taking the remedy so indicated, comes in, there is no need to go over the study of the case again, because we have the whole of the previous study mapped out and ready for immediate reference. Thus this book is a time-saver. We have, as before stated, made frequent use of it in difficult cases, and find it works very satisfactorily.

